# Efficacy and safety of bone marrow aspirate and bone marrow aspirate concentrate in orthopedic conditions: a systematic review of randomized controlled trials

**DOI:** 10.3389/fsurg.2026.1932090

**Published:** 2026-10-02

**Authors:** Joelington Dias Batista, Ludimila Dias Silva, Jobson Dias Batista, Gabrielly Santos Pereira, Marcelo Lourenço da Silva

**Affiliations:** 1 Orthopain Pain Institute, Anápolis, Brazil; 2 Neuropsychobiology and Motor Behavior Laboratory, Faculty of Medicine of Ribeirão Preto, University of São Paulo – USP, Ribeirão Preto, Brazil; 3 Laboratory of Neuroscience, Neuromodulation and Study of Pain (LANNED), Federal University of Alfenas (UNIFAL-MG), Alfenas, MG, Brazil

**Keywords:** bone marrow aspirate, bone marrow aspirate concentrate, cartilage repair, ligament injury, orthobiologics, osteoarthritis, randomized controlled trials

## Abstract

**Introduction:**

Bone marrow aspirate (BMA) and bone marrow aspirate concentrate (BMAC) have emerged as autologous orthobiologic strategies aimed at modulating inflammation and promoting tissue repair in orthopedic conditions. However, evidence from randomized controlled trials (RCTs) remains heterogeneous, and their clinical role relative to established treatments is still debated. This systematic review aimed to evaluate the efficacy, structural effects, and safety of BMA and BMAC in adults with orthopedic conditions based exclusively on RCT evidence.

**Methods:**

A systematic review was conducted according to PRISMA 2020 guidelines. PubMed, Embase, Scopus, and Web of Science were searched from December 2015 to December 2025. Eligible studies were RCTs comparing BMA or BMAC with placebo, conventional injective therapies, exercise therapy, or standard surgical procedures. Outcomes included pain, functional performance, imaging-based structural healing, treatment failure or revision, and safety. Risk of bias was assessed using the Cochrane RoB 2.0 tool, and results were synthesized narratively.

**Results:**

Twenty-one RCTs involving knee osteoarthritis, ACL injury or reconstruction, rotator cuff tears, focal cartilage defects, osteonecrosis, discogenic low back pain, and glenohumeral osteoarthritis were included. Across trials, BMA/BMAC was generally associated with improvements in pain and function, although superiority over comparators was inconsistent and depended on the clinical condition and treatment strategy.

**Discussion:**

Signals of greater benefit were observed in selected patients with early-stage knee osteoarthritis, focal cartilage pathology, nonsurgical ligament injuries, and rotator cuff disorders.

**Systematic Review Registration:**

https://www.crd.york.ac.uk/PROSPERO/view/CRD420251241519, identifier CRD420251241519.

## 1 Introduction

Orthopedic and musculoskeletal disorders represent one of the leading causes of chronic pain, functional disability, and loss of productivity in adults, with a growing impact on healthcare systems driven by population aging and increased participation in sports and physical activity (1, 2). Conditions such as osteoarthritis, ligament and tendon injuries, osteochondral defects, osteonecrosis, and degenerative low back pain share common pathophysiological mechanisms, including persistent inflammation, disruption of tissue homeostasis, and a limited intrinsic capacity for regeneration (3). Although conventional conservative and surgical approaches are widely employed, their outcomes are often variable, which has stimulated increasing interest in bone marrow aspirate–based therapies as regenerative biological strategies aimed at modulating the local tissue environment and stimulating endogenous repair mechanisms (4, 5).

In these conditions, the initial tissue insult triggers a physiological cascade of damage responses involving inflammatory processes and nociceptor activation secondary to cellular and structural injury (6). Disruption or degeneration of the extracellular matrix and resident cells activates early inflammatory pathways, leading to the release of mediators such as prostaglandins and pro-inflammatory cytokines, including interleukin-1β (IL-1β) and tumor necrosis factor-α (TNF-α) (7, 8). These mediators are associated with increased production of tissue-degrading enzymes and activation of catabolic pathways. The resulting vasodilation, increased vascular permeability, and immune cell recruitment promote local edema and peripheral nociceptor sensitization, amplifying pain transmission (9). When tissue regeneration is insufficient and the injurious stimulus persists, inflammation becomes chronic, contributing to peripheral and central sensitization, whereby previously non-noxious mechanical or chemical stimuli elicit pain due to reduced nociceptor activation thresholds and neural circuit reorganization (10). Concurrently, progressive structural disorganization—such as cartilage thinning, tendon fissuring, subchondral bone alterations, or intervertebral disc degeneration—perpetuates mechanical instability, sustains chronic inflammation, and maintains the cycle of pain and functional impairment (3).

Within this context, orthobiologic therapies have emerged as promising strategies aimed at modulating the local biological milieu, stimulating endogenous repair processes, and potentially altering the natural history of musculoskeletal disorders (11). Autologous biologic products have increasingly been investigated for their capacity to influence inflammatory responses and promote tissue repair in musculoskeletal injuries (12). Among these approaches, bone marrow aspirate (BMA) and bone marrow aspirate concentrate (BMAC) have received growing attention due to their content of cells with regenerative potential and bioactive signaling molecules capable of interacting with injured tissues (13). These autologous preparations contain heterogeneous cell populations, including mesenchymal progenitor cells, along with cytokines, growth factors, and immunomodulatory components that may act synergistically to support bone, cartilage, ligament, and tendon repair through mechanisms such as chondrogenesis, osteogenesis, and modulation of inflammatory pathways (14).

In general, bone marrow aspirate–based therapy relies on the autologous harvest of cellular and molecular components from the bone marrow—most commonly obtained from the iliac crest—which can be administered as whole aspirate (BMA) or after concentration (BMAC) (15). These biologic products are applied directly to the site of injury with the objective of creating a biologically favorable environment for tissue repair through inflammation modulation, trophic factor release, and stimulation of endogenous regenerative processes. Importantly, this approach seeks not only symptomatic relief but also modulation of the underlying structural mechanisms involved in the progression of musculoskeletal disease (16).

Preclinical and early clinical studies suggest that BMA and BMAC may exert analgesic, anti-inflammatory, and pro-regenerative effects, influencing both clinical outcomes—such as pain and function—and structural markers assessed by imaging modalities (17, 18). However, the clinical literature remains highly heterogeneous with respect to indications, preparation techniques, delivery routes, biological dosing, and comparator interventions. Moreover, reported outcomes are inconsistent across different orthopedic conditions, with some trials demonstrating meaningful clinical and structural benefits, while others fail to show superiority over placebo, conventional injectable therapies, or standard surgical procedures (19, 20).

Given this variability, a critical and systematic synthesis of the available evidence is necessary to integrate data on clinical efficacy, imaging-based tissue regeneration, and treatment safety, as well as to identify subgroup effects and methodological gaps. Therefore, the aim of this systematic review was to comprehensively evaluate the effects of BMA and BMAC in adults with orthopedic conditions, examining their impact on pain, functional outcomes, structural healing, and safety across different clinical contexts and therapeutic strategies currently described in the literature.

## 2 Methods

### 2.1 Study design

This systematic review examined the effects of bone marrow aspirate (BMA) and bone marrow aspirate concentrate (BMAC) on pain, functional outcomes, structural tissue regeneration, and treatment safety in adults with orthopedic conditions. The review included randomized controlled trials evaluating the short- and long-term clinical efficacy, imaging biomarkers of healing, and safety of BMA/BMAC interventions. The review followed PRISMA 2020 guidelines and was prospectively registered in PROSPERO (CRD420251241519).

### 2.2 Eligibility criteria

Eligibility criteria were defined using the PICO framework.

#### 2.2.1 Population

Adults (≥18 years) diagnosed with orthopedic or musculoskeletal conditions, including knee osteoarthritis, osteochondral lesions, anterior cruciate ligament (ACL) injury or reconstruction, rotator cuff tears, meniscus tears, osteonecrosis of the femoral head, chronic low back pain, femoroacetabular impingement, and upper-limb nonunion. Studies involving pediatric populations, preclinical models, cultured or genetically modified cells, or mixed samples without extractable BMA/BMAC-specific outcome data were excluded.

#### 2.2.2 Intervention

Autologous bone marrow aspirate (BMA) or bone marrow aspirate concentrate (BMAC) administered through intra-articular, intraosseous, subchondral, intradiscal, percutaneous tendon/ligament injection, or used as biologic augmentation during orthopedic surgery (e.g., ACL grafts, osteochondral allografts, rotator cuff repair). Both single-injection and multi-injection protocols, as well as combination approaches such as BMAC plus platelet-rich plasma (PRP), were eligible.

#### 2.2.3 Comparison

Eligible comparators included placebo or saline injection, hyaluronic acid, platelet-rich plasma, corticosteroid injections, standard postoperative or conservative care, exercise therapy, or surgical procedures without biologic augmentation.

#### 2.2.4 Primary outcomes

Pain reduction assessed using validated measures such as the Visual Analog Scale (VAS), Numeric Pain Scale (NPS), or pain-specific subscores of WOMAC or KOOS, including rest pain, movement-evoked pain, and postoperative pain.

#### 2.2.5 Secondary outcomes

Functional outcomes such as WOMAC total score, KOOS, IKDC, Lysholm, DASH, ODI, SPADI; structural healing evaluated by MRI, CT, or x-ray (cartilage fill, graft integration, ligamentization, bone marrow lesion resolution); activity measures; treatment failure or revision surgery; and adverse events including donor-site morbidity, local inflammatory reactions, procedural complications, or neurological symptoms.

#### 2.2.6 Study designs

Eligible designs included randomized controlled trials (double-blind, single-blind, or open-label). Case reports, cohort studies, comparative observational studies, small case series, and conference abstracts without extractable data were excluded. Studies were eligible when BMA/BMAC constituted a predefined component of the randomized intervention, including combination biologic protocols and surgical augmentation strategies. Studies in which the contribution of BMA/BMAC could not be identified as part of the randomized treatment strategy were excluded.

### 2.3 Information sources

Electronic searches were conducted in PubMed, Scopus, Embase, and Web of Science, covering December 2015 to December 2025, English language restrictions. Additional sources included reference lists, forward citation tracking, and manual searches.

### 2.4 Search strategy

The search strategy combined MeSH terms and free-text keywords related to bone marrow aspirate, bone marrow aspirate concentrate, orthobiologics, and orthopedic conditions. Boolean operators were used to capture studies evaluating biologic injections or surgical augmentation with BMA/BMAC in musculoskeletal disorders. Example terms included: “bone marrow aspirate,” “bone marrow aspirate concentrate,” “BMAC,” “orthopedic,” “osteochondral,” “rotator cuff,” “ACL,” “meniscus,” “osteonecrosis,” and “randomized trial.” Screening decisions incorporated both lexical and contextual relevance.

### 2.5 Study selection

All records were imported into Rayyan for duplicate removal and screening. Two independent reviewers (M.L.S. and G.S.P.) screened titles/abstracts and full texts according to prespecified criteria. Disagreements were resolved through discussion and, when consensus could not be reached, by adjudication by a third reviewer (J.D.B.). A PRISMA 2020 flow diagram summarizes the selection process (Figure 1).

**Figure 1:**
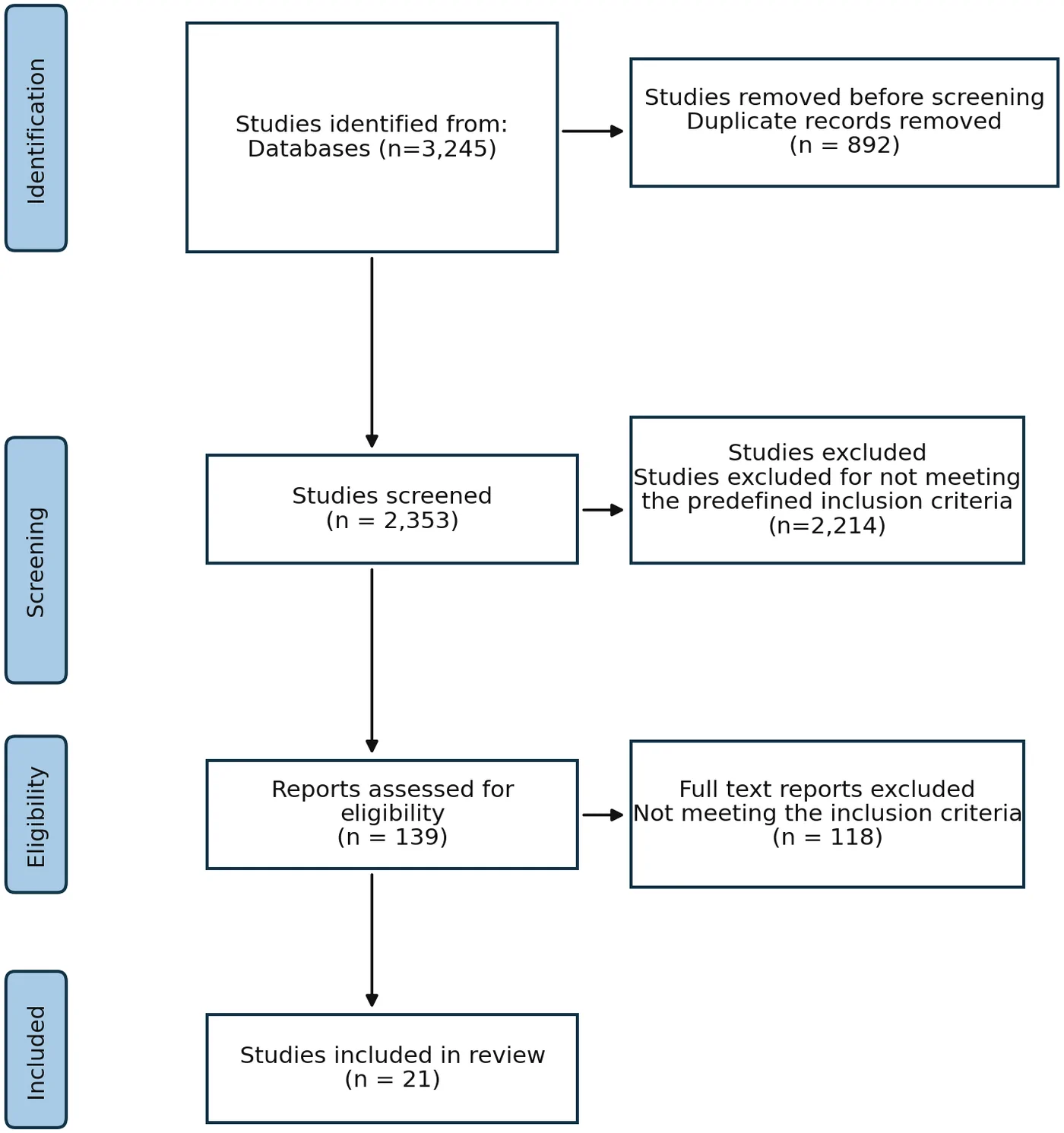
PRISMA-style flowchart showing study selection for a review; three thousand two hundred forty-five identified, eight hundred ninety-two removed as duplicates, two thousand three hundred fifty-three screened, two thousand two hundred fourteen excluded, one hundred thirtynine assessed, one hundred eighteen excluded, and twentyone included in the final review.

### 2.6 Data extraction

Data were extracted using a standardized template capturing study characteristics (design, blinding, sample size, follow-up), participant characteristics and demographics (age, sex, body mass index [BMI], clinical condition, disease severity, and baseline pain and functional status), BMA/BMAC preparation characteristics (aspirate volume, concentration method, cell counts when available), delivery method (intra-articular, subchondral, intradiscal, surgical augmentation), comparators, pain and functional outcomes, imaging biomarkers, revision or failure rates, and adverse events. Subgroup variables such as disease severity, timing of treatment, smoking status, biological dose, and presence of concurrent pathology were also extracted when reported. Extracted information was reviewed for accuracy and consistency.

### 2.7 Risk of bias assessment

Risk of bias for randomized controlled trials was evaluated using the Cochrane Risk of Bias 2.0 tool, examining sequence generation, allocation concealment, blinding, deviations from intended interventions, completeness of outcome data, measurement reliability, and selective reporting. Graphical summaries were generated using ROBVIS.

### 2.8 Data synthesis

Given the substantial heterogeneity in study populations, biologic preparation methods, delivery approaches, comparators, and outcome metrics, results were synthesized narratively. Studies were grouped according to clinical condition (e.g., knee osteoarthritis, ACL reconstruction, rotator cuff tears, osteonecrosis), intervention approach (injection vs. surgical augmentation), and outcome domain (pain, function, imaging biomarkers, safety). Emphasis was placed on identifying consistent patterns of clinical benefit, imaging evidence of tissue regeneration, and subgroup effects related to disease severity, comorbid pathology, and biological dose–response relationships.

## 3 Results

### 3.1 Study selection and characteristics

The systematic search yielded 3,245 records. After removal of 892 duplicates, 2,353 titles and abstracts were screened for eligibility. Of these, 2,214 were excluded for not meeting inclusion criteria, leaving 139 full-text articles for detailed assessment. After full-text review, 21 studies met all eligibility criteria and were included in this review (Figure 1).

These trials encompassed conditions such as knee osteoarthritis, ACL reconstruction, ACL tears treated non-surgically, rotator cuff tears, osteochondral lesions, osteonecrosis, and glenohumeral osteoarthritis. Follow-up duration ranged from 3 months (0.25 years) to 15 years, allowing assessment of both short-term clinical responses and long-term durability of BMA/BMAC-related outcomes. Across trials, interventions involved intra-articular, intraosseous, subchondral, intradiscal, or intra-tendon/peri-graft application of BMAC, while comparators included saline, hyaluronic acid (HA), platelet-rich plasma (PRP), corticosteroids, exercise therapy, or standard surgical procedures without biologic augmentation (Table 1).

**Table 1:** Characteristics and quantitative findings of randomized trials evaluating BMA/BMAC in orthopedic conditions.

Study	Condition/design	Sample size	Baseline demographics*	Intervention	Comparator	Follow-up/outcomes	Quantitative key findings
Forsythe et al. (21)	ACL reconstruction with BTB allograft; double-blind RCT	80 randomized; 73 analyzed at 1 y (BMAC 36; control 37)	Age: 36.3 ± 9.5 vs. 36.6 ± 8.8 y; male: 38.5% vs. 42.9%; BMI: 27.9 ± 7.1 vs. 27.6 ± 4.6	BMAC augmentation of BTB allograft	Standard ACLR + sham harvest incision	MRI 3/9 mo; clinical ≤24 mo; SIR, IKDC, KOOS-JR, Tegner	Inferior-third graft SIR was higher with BMAC at 3 mo (3.2 ± 2.2 vs. 2.1 ± 1.5; *P* = .023), consistent with greater early remodeling activity. IKDC was higher at 9 mo (81.6 ± 10.5 vs. 74.6 ± 14.2; *P* = .048), but the 7-point difference did not exceed the established MCID; MCID achievement was 89% vs. 85% (*P* = .70).
Boffa et al. (22)	Bilateral knee OA; double-blind paired-knee RCT	60 enrolled; 56 completed (112 knees)	Age: 57.8 ± 8.9 y; 35M/21F; BMI: 27.8 ± 4.3	IA BMAC	Contralateral HA	24 mo; VAS, IKDC, KOOS, radiographs	Overall clinical results were largely comparable, but VAS improvement favored BMAC at 12 mo (Δ − 2.2 ± 2.6 vs. −1.7 ± 2.5; *P* = .041) and 24 mo (Δ − 2.2 ± 2.6 vs. −1.4 ± 2.8; *P* = .002). The advantage was greater in KL 1–2 OA; no clear radiographic superiority was demonstrated.
Shapiro et al. (23)	Bilateral knee OA; single-blind placebo-controlled paired-knee RCT	25 patients/50 knees	Age: median 60 y (42–68); 7M/18F; BMI: median 27.1 (18.4–37.5)	IA BMAC	Contralateral saline	6 mo; ICOAP, VAS, algometry	Pain improved substantially in both BMAC- and saline-treated knees, without significant between-knee differences in ICOAP (*P* > .09) or VAS change (*P* > .44). No major safety signal was identified.
Shapiro et al. (24)	Bilateral knee OA; 12-mo report of same paired-knee RCT	25 patients/50 knees	Same cohort as Shapiro et al. (23)	IA BMAC	Contralateral saline	12 mo; ICOAP, VAS, activity, KL grade, quantitative T2 MRI	Pain improvement persisted in both knees, with no significant BMAC-placebo differences (all pain comparisons *P* ≥ .23). No meaningful advantage in radiographic OA progression or quantitative cartilage T2 measures was demonstrated.
Yanke et al. (25)	Osteochondral allograft transplantation; prospective double-blind RCT	37 randomized; 36 analyzed (BMAC 19; control 17)	Age: 34.3 ± 9.5 vs. 29.9 ± 8.8 y; M/F: 6/13 vs. 13/4; BMI: 27.7 ± 5.2 vs. 27.7 ± 4.2	BMAC-treated OCA graft	Standard OCA + sham incision	CT 6 mo; PROMs ≤2 y; CT integration, IKDC, KOOS-JR, reoperation	Osseous integration did not differ significantly (*P* = .49). Subsequent surgery was less frequent with BMAC (5.3% vs. 35.3%; *P* = .023). Small cysts were more frequent with BMAC (*P* = .01), whereas large cyst formation showed a favorable trend (*P* = .06). PROMs did not significantly differ between groups.
Lin et al. (26)	ACL reconstruction; prospective double-blind 3-arm RCT	30 randomized; 27 completed (control 10; PRP 8; BMAC + PRP 9)	Age: 29.7 ± 9.9/28.4 ± 7.8/27.1 ± 3.9 y; M/F: 6/4, 6/2, 5/4; BMI: 24.7 ± 4.7/25.0 ± 4.1/24.3 ± 2.8	BMAC + PRP augmentation	PRP alone or standard ACLR	48 wk; IKDC, Lysholm, KT-1000, MRI	BMAC + PRP provided no significant functional-score advantage. Knee laxity showed limited improvement at 24 wk, while early MRI demonstrated greater graft/peri-tunnel signal and tunnel changes; femoral tunnel diameter at 24 wk was greater vs. control (*P* = .024) and PRP (*P* = .008).
Centeno et al. (27)	Nonretracted supraspinatus tears; randomized crossover RCT, interim report	25 enrolled (BMC 14; exercise 11)	Age: 46 ± 11 vs. 49 ± 11 y; M/F: 9/5 vs. 5/6; BMI: 26.8 ± 3.7 vs. 25.9 ± 5.3	Percutaneous BMC + PRP + PL	Home exercise therapy	≤24 mo; DASH, NPS, SANE, MRI	DASH favored BMC at 3 mo (*P* < .05) and approached significance at 6 mo (*P* = .06); NPS favored BMC at 3 and 6 mo and SANE at 3 mo. Mean SANE reached 80% at 12 mo and 89% at 24 mo. Post-treatment MRI was judged healthier in 14/15 paired scans; mean tear-size reduction was 26% but was not statistically significant.
Anz et al. (28)	Knee OA; prospective randomized BMC-vs.-PRP trial	90 randomized; 84 included in analyzed dataset (BMC 45; PRP 39)	Age: 55.8 ± 11.3 vs. 52.2 ± 12.4 y; M/F: 27/18 vs. 22/17; BMI: 27.7 ± 5.0 vs. 27.9 ± 5.8	IA BMC	Leukocyte-rich PRP	24 mo; IKDC, WOMAC	Both groups improved by 1 mo, with approximately maximal improvement by 3 mo and maintenance through 24 mo. No BMC superiority was observed for IKDC (*P* = .909) or WOMAC total (*P* = .789). At 24 mo, IKDC improvement was approximately +16.6 vs. +16.0 points and WOMAC improvement 14.5 vs. 12.4 points.
Cole et al. (29)	Arthroscopic rotator cuff repair; prospective single-blind RCT	91 randomized (cBMA 46; control 45); 82 completed ≥2 y; 75 had 1-y MRI	Age: 56.1 ± 10.1 vs. 55.3 ± 9.6 y; M/F: 25/21 vs. 32/13; BMI: 28.9 ± 5.6 vs. 30.4 ± 5.5	cBMA augmentation of aRCR	Standard aRCR + sham hip incision	Clinical 24 mo; MRI 12 mo; ASES, SANE, SST, MRI Sugaya	PROMs improved similarly in both groups. Retear on 12-mo MRI (Sugaya 4–5) was 18% with cBMA vs. 57% control (*P* < .001); mean Sugaya grade was 2.63 ± 0.91 vs. 3.43 ± 1.04 (*P* < .001). Overall treatment failure remained similar (15% vs. 16%; *P* = .964).
de Girolamo et al. (30)	Focal knee chondral lesions; randomized controlled trial	24 randomized (12/12); 16 available at 100 mo (BMAC 9; control 7)	Age: 30.0 ± 11.3 vs. 30.0 ± 10.2 y; M/F: 8/4 vs. 7/5; BMI: NR	AMIC + BMAC	AMIC alone	100 mo; VAS, Lysholm, IKDC, Tegner, KOOS, MRI	At 12 mo, AMIC + BMAC showed lower VAS (*P* = .011) and higher Lysholm (*P* = .015). Both groups maintained substantial clinical improvement to 100 mo, but later between-group outcomes were broadly comparable; MRI showed satisfactory repair in both groups.
Centeno et al. (31)	KL II–III knee OA; randomized crossover RCT	48 randomized (BMC 26; exercise 22); BMC *n* = 24 available at randomized 3-mo comparison	Age: 54 ± 8.9 vs. 57 ± 8.5 y; sex: NR; BMI: 26 ± 2.9 vs. 26 ± 2.9	Image-guided BMC + platelet-product protocol	Home exercise therapy	24 mo; randomized comparison through 3 mo; KSS, VAS, SF-12, LEAS, ROM	At 3 mo, BMC produced greater improvement in KSS-knee (*P* < .001) and LEAS (*P* < .01); VAS, KSS-function, SF-12, and ROM did not significantly differ. All 22 exercise patients subsequently crossed over, preventing randomized long-term between-group comparison.
Dwyer et al. (32)	Glenohumeral OA; randomized pilot trial	22 patients/25 shoulders completing 12 mo (BMA 13; corticosteroid 12)	Age: 61.6 vs. 53.8 y (*P* = .021); sex/BMI: NR in extractable study table	10 mL IA nonconcentrated BMA	IA corticosteroid	12 mo; WOOS, QuickDASH, EQ-5D, VAS	BMA showed significant within-group improvement in WOOS (*P* = .002) and QuickDASH (*P* < .001). Between-group change did not reach significance for the primary WOOS endpoint (*P* = .07), but favored BMA for QuickDASH (*P* = .006), EQ-5D pain (*P* = .003), and EQ-5D health (*P* = .032). No imaging outcome was reported.
Hernigou et al. (33)	Bilateral knee OA; prospective randomized paired-knee study	140 patients/280 knees	Age: 75.4 y (65–90); 53M/87F; BMI: 28.1 (19.5–32.4)	Subchondral BMC injection	Contralateral TKA	Mean 15 y (10–20); VAS, KSS, MRI BML, conversion to TKA	BML volume decreased by a mean ≈1.5 cm^3^ over the first 24 mo. Twenty-five of 140 BMC-treated knees (18%) ultimately underwent TKA at a mean ≈10 y. Arthroplasty incidence after BMC was 1.19% patient-year vs. contralateral TKA revision 1.00% patient-year (*P* = .34). Persistent BML >3 cm^3^ at 2 y predicted later TKA (HR 4.42, 95% CI 2.34–7.21; *P* < .001).
Clazzer et al. (34)	Knee OA; prospective single-blind RCT	50 randomized (25/25); 35 evaluated at 6 mo (BMA 17; control 18)	Age: ≈58.1 vs. 57.8 y; M/F at 6 mo: 5/12 vs. 5/13; BMI: NR	IA BMA within multimodal injection/block protocol	Corticosteroid + genicular nerve block	6 mo; WOMAC pain, stiffness, physical function	At 6 mo, WOMAC pain favored BMA (*P* = .030); differences in stiffness (*P* = .097) and physical function (*P* = .114) were not significant. Thus, evidence favored BMA for pain but did not demonstrate clear global functional superiority.
Hauzeur et al. (35)	ARCO stage 3 osteonecrosis of femoral head; double-blind RCT	52 hips/42 patients randomized; 46 hips/38 patients analyzed (23 hips/group)	Age: 48.0 ± 2.8 vs. 49.7 ± 3.2 y; M/F: 14/5 vs. 13/6; BMI: 25.35 ± 0.75 vs. 24.53 ± 0.96	Core decompression + BMAC	Core decompression + saline	24 mo; THR, VAS, WOMAC, radiographic progression	BMAC produced no significant benefit for the primary THR endpoint (*P* = 1.000), time to THR (9.7 ± 1.2 vs. 8.2 ± 0.8 mo; *P* = .382), clinical outcomes, or progression to ARCO stage 4 (*P* = .871). Results do not support an added effect of BMAC in established stage-3 disease.
Navani et al. (36)	Discogenic chronic low back pain; multicenter prospective crossover RCT	43 treated/completing trial; 40 with complete 12-mo data (placebo 12; PRP 13; BMC 15)	Age at 12 mo: 44.8 ± 11.0/47.1 ± 10.5/44.1 ± 7.3 y; M/F: 6/6, 6/7, 6/9; BMI: NR	Intradiscal BMC	Intradiscal PRP and saline/placebo protocol	12 mo; NRS, ODI, NASS	Both PRP and BMC reduced NRS significantly compared with placebo over 12 mo (*P* < .001), with no significant BMC-vs.-PRP difference. ODI improvement remained significant vs. baseline at 12 mo for PRP (*P* = .0144) and BMC (*P* = .0109); PRP vs. BMC *P* = .30.
Centeno et al. (37)	Non-surgical ACL tears; randomized crossover RCT	51 randomized (BMC 34; exercise 17); 3 exercise patients withdrew before treatment	Age: 36.8 ± 10.6 vs. 40.9 ± 9.5 y; M/F: 25/9 vs. 7/7 among treated exercise patients; BMI: 24.2 ± 2.7 vs. 24.8 ± 4.0	Intraligament BMC + PRP + PL	Exercise therapy	24 mo; IKDC, LEFS, NPS, SANE, MRI	At 3 mo, ΔLEFS was +9 [IQR 1–14] vs. +1 [−2.5–4.75] (*P* = .027) and SANE 60% [42.5–77.5] vs. 20% [0–50] (*P* = .007). ΔIKDC (10.4 vs. 5.7) and ΔNPS (−1 vs. −1) did not differ significantly. MRI ACL signal intensity decreased after BMC (*P* < .001). Four patients ultimately underwent ACLR.
Centeno et al. (38)	High-grade partial/full-thickness nonretracted supraspinatus tears; randomized crossover RCT, final report	51 randomized (BMC 34; exercise 17)	Exercise: 8F/9M; age F52 ± 11/M47 ± 12; BMI F24.9 ± 5.4/M28.3 ± 6.1. BMC: 10F/24M; age F54 ± 11/M44 ± 12; BMI F24.8 ± 3.2/M26.4 ± 3.2	Percutaneous BMC + PRP + PL	Home exercise therapy	24 mo; DASH, NPS, SANE, MRI	At 3 mo, median ΔDASH was −11.7 vs. −3.8 (*P* = .01), ΔNPS −2.0 vs. +0.5 (*P* = .004), and SANE 50 vs. 0 (*P* < .001). At ≥1 y, 27/37 (73%) paired MRIs showed improved tear severity/location. PROM improvement persisted through 24 mo; one patient required RC surgery.
Cole et al. (39)	Arthroscopic rotator cuff repair; preliminary randomized single-blind report	62 randomized (BMAC 28; control 34)	Age/sex/BMI: NR in preliminary abstract	cBMA/BMAC augmentation of aRCR	Standard repair + sham harvest procedure	Clinical ≤24 mo; MRI 12 mo; SST, ASES, Constant, SANE, MRI Sugaya	Clinical outcomes were similar (12-mo SST 87.50 vs. 85.32, *P* = .18; ASES 87.38 vs. 89.96, *P* = .67). MRI favored biologic augmentation: mean Sugaya 2.60 ± 0.91 vs. 3.25 ± 1.12; overall Sugaya distribution differed significantly (*P* = .0012), although the mean-grade comparison was *P* = .067.
Mautner et al. (40)	Knee OA; multicenter single-blind phase 2/3 randomized trial	480 randomized; 440 received study intervention/included in primary analyses	BMAC cohort: age ≈58.6 ± 7.3 y; ≈56M/62F; BMI ≈30.6 ± 6.0	IA BMAC	Corticosteroid, adipose-derived SVF, or allogeneic UCT-MSC	12 mo; VAS, KOOS pain, MRI	No orthobiologic treatment demonstrated superiority to corticosteroid at 1 y. For BMAC vs. corticosteroid, VAS change was approximately −24.3 vs. −20.9 (*P* = .19) and KOOS-pain improvement +19.1 vs. +17.7 (*P* = .49). No meaningful MRI structural advantage was demonstrated. There was no PRP arm in this trial.
Anz et al. (41)	ACL autograft reconstruction; prospective single-blind pilot RCT, stratified by graft type	40 randomized: BTB 20 and HS 20; each stratum 10 intervention/10 control	Age range 18–35 y; BTB control 29.0 ± 6.8 vs. intervention 29.8 ± 8.2; BTB M/F 5/5 vs. 6/4; BMI: NR	Amnion collagen matrix wrap + BMAC	Standard ACLR	24 mo; MRI T2*, graft volume, SANE, KOOS, VAS	No significant augmentation effect was found for BTB T2* (95% CI −1.89 to 0.63; *P* = .31), BTB graft volume (*P* = .71), HS T2* (*P* = .162), or HS graft volume (*P* = .28). No significant differences occurred in PROMs, and no adverse events were reported through 2 y.

* **Baseline demographics:** unless otherwise specified, values are presented as intervention vs. comparator and as mean ± SD. For three-arm studies, values follow the order in which treatment groups are listed. NR, not reported or not extractable from the publication. BMA, bone marrow aspirate; BMAC/BMC, bone marrow aspirate concentrate/bone marrow concentrate; PL, platelet lysate; PRP, platelet-rich plasma; HA, hyaluronic acid; IA, intra-articular; ACL(R), anterior cruciate ligament (reconstruction); BTB, bone–patellar tendon–bone; OCA, osteochondral allograft; OA, osteoarthritis; AMIC, autologous matrix-induced chondrogenesis; BML, bone marrow lesion; THR, total hip replacement; TKA, total knee arthroplasty; SVF, stromal vascular fraction; UCT-MSC, umbilical cord tissue-derived mesenchymal stromal cells; MCID, minimal clinically important difference.

### 3.2 Pain outcomes

Pain outcomes varied according to the clinical condition and comparator, with improvements frequently observed in both BMA/BMAC and control groups. In knee osteoarthritis, Boffa et al. (22) reported greater VAS improvement with BMAC than with hyaluronic acid at 12 months (Δ − 2.2 ± 2.6 vs. Δ − 1.7 ± 2.5; *P* = .041) and 24 months (Δ − 2.2 ± 2.6 vs. Δ − 1.4 ± 2.8; *P* = .002), with the greatest between-group differences observed in patients with Kellgren–Lawrence grade 1–2 disease. In contrast, the placebo-controlled trials by Shapiro et al. (23, 24) demonstrated significant pain improvement within both BMAC- and saline-treated knees, without significant between-treatment differences at 6 months (ICOAP, *P* > .09; VAS, *P* > .44) or 12 months (all between-treatment pain comparisons, *P* ≥ .23). In the trial by Anz et al. (28), improvement after BMC and PRP was already evident during the first months and was maintained through 24 months, without significant superiority of BMC. Similarly, in the multicenter trial by Mautner et al. (40), the 12-month VAS change was −24.3 with BMAC vs. −20.9 with corticosteroid (*P* = .19), indicating clinically relevant improvement but no significant between-group superiority.

In rotator cuff pathology, Centeno et al. (27, 38) reported an earlier analgesic effect with BMC combined with platelet products compared with exercise therapy. In the final randomized cohort, the median change in NPS at 3 months was −2.0 with BMC vs. +0.5 with exercise (*P* = .004), and clinical improvements were maintained through 24 months.38 In surgical rotator cuff repair, Cole et al. (29, 39) found substantial symptomatic improvement in both groups without a significant additional patient-reported analgesic benefit from cBMA augmentation. In glenohumeral osteoarthritis, Dwyer et al. (32) reported that the between-group change favored BMA for the EQ-5D pain domain (*P* = .003), although the primary WOOS comparison did not reach statistical significance (*P* = .07). Pain outcomes following ACL reconstruction were generally comparable between augmented and control groups (21, 26, 41), whereas in non-surgical ACL tears the 3-month change in NPS was similar between BMC and exercise (−1 vs. −1) (37).

### 3.3 Functional outcomes

Functional outcomes also varied according to indication and comparator. In knee osteoarthritis, Boffa et al. (22) reported broadly comparable functional outcomes between BMAC and hyaluronic acid, despite the greater VAS improvement observed with BMAC at later follow-up. In the BMC-vs.-PRP trial by Anz et al. (28), both groups demonstrated sustained functional improvement through 24 months. At 24 months, IKDC improvement was approximately +16.6 points with BMC vs. +16.0 points with PRP, while WOMAC improvement was 14.5 vs. 12.4 points, respectively; neither IKDC (*P* = .909) nor WOMAC (*P* = .789) differed significantly between groups. In the crossover trial by Centeno et al. (31), BMC produced greater improvement than exercise at 3 months in KSS-knee (*P* < .001) and LEAS (*P* < .01), although VAS, KSS-function, SF-12, and range of motion did not significantly differ between groups.

In ACL reconstruction, Forsythe et al. (21) reported a higher IKDC score with BMAC augmentation at 9 months (81.6 ± 10.5 vs. 74.6 ± 14.2; *P* = .048), although the 7-point between-group difference did not exceed the established MCID and MCID achievement was similar between groups (89% vs. 85%; *P* = .70). In non-surgical ACL tears, Centeno et al. (37) found greater 3-month improvement in LEFS with BMC than with exercise (+9 [IQR 1–14] vs. +1 [IQR −2.5 to 4.75]; *P* = .027) and higher SANE scores (60% [42.5–77.5] vs. 20% [0–50]; *P* = .007), whereas the change in IKDC (10.4 vs. 5.7) was not significantly different.

In rotator cuff pathology, Centeno et al. (38) reported a greater median reduction in DASH at 3 months with BMC than with exercise (Δ − 11.7 vs. Δ − 3.8; *P* = .01), together with higher SANE scores (50 vs. 0; *P* < .001), with improvement persisting through 24 months. Conversely, surgical trials by Cole et al. (29, 39) showed substantial functional improvement in both augmented and control groups without significant between-group differences in patient-reported outcomes. In focal cartilage repair, de Girolamo et al. (30) reported higher Lysholm scores with AMIC + BMAC at 12 months (*P* = .015), whereas both groups maintained substantial clinical improvement through approximately 100 months. In advanced osteonecrosis, Hauzeur et al. (35) found no significant functional advantage of BMAC over core decompression plus saline at 24 months.

### 3.4 Imaging and structural outcomes

Structural and imaging outcomes varied substantially across indications. In ACL reconstruction, Forsythe et al. (21) reported a higher signal intensity ratio in the inferior third of the graft at 3 months in the BMAC group (3.2 ± 2.2 vs. 2.1 ± 1.5; *P* = .023), indicating greater early graft remodeling activity. However, this imaging difference was not accompanied by a clear long-term structural advantage. Lin et al. (26) found no significant between-group difference in whole-graft maturation, although the BMAC + PRP group showed a later graft-signal peak and a larger femoral tunnel diameter at 24 weeks than both the control (*P* = .024) and PRP groups (*P* = .008). Similarly, Anz et al. (41) found no significant augmentation effect on BTB graft T2 (*P* = .31), BTB graft volume (*P* = .71), hamstring graft T2 (*P* = .162), or hamstring graft volume (*P* = .28).

In non-surgical ACL tears, Centeno et al. (37) reported a significant reduction in MRI ACL signal intensity following BMC treatment (*P* < .001), consistent with structural changes during healing. In osteochondral allograft transplantation, Yanke et al. (25) found no significant difference in osseous integration between BMAC-treated and control grafts at 6 months (*P* = .49), although subsequent surgery was less frequent after BMAC treatment (5.3% vs. 35.3%; *P* = .023). In knee osteoarthritis, Shapiro et al. (24) found no significant improvement in quantitative T2 cartilage mapping after BMAC compared with saline, providing no MRI evidence of cartilage regeneration following a single injection.

In rotator cuff pathology, Centeno et al. (27, 38) reported MRI evidence of improved tear morphology following BMC combined with platelet products. In the interim analysis, 14 of 15 paired MRI examinations were judged to show a healthier tendon after treatment, although the mean 26% reduction in tear size was not statistically significant.27 In the final report, 27 of 37 patients (73%) with paired MRI examinations demonstrated improvement in tear severity or location (38). The strongest structural effect was observed in the randomized surgical trial by Cole et al. (29), in which full-thickness retears on 12-month MRI occurred in 18% of cBMA-augmented repairs compared with 57% of controls (*P* < .001); mean Sugaya grade was also lower with cBMA (2.63 ± 0.91 vs. 3.43 ± 1.04; *P* < .001).

In advanced osteonecrosis of the femoral head, BMAC did not demonstrate structural superiority over core decompression plus saline in ARCO stage 3 disease (35). Progression to ARCO stage 4 and the requirement for total hip replacement were similar between groups, indicating limited structural efficacy once femoral head collapse was already established.

### 3.5 BMA/BMAC preparation and processing heterogeneity

Substantial methodological heterogeneity was observed across the included randomized trials regarding bone marrow harvesting, processing, and delivery protocols. Some studies utilized minimally processed BMA, in which aspirated bone marrow was injected without additional processing, whereas others applied centrifugation-based concentration systems to produce BMAC, aiming to enrich the mononuclear cell fraction and growth factor content (21–24, 28, 40).

Centrifugation protocols and processing systems varied considerably across trials. Several studies employed commercial centrifugation systems designed to concentrate mesenchymal stromal cells and progenitor cells prior to injection (21, 22, 28, 40), whereas others used simpler preparation approaches or did not provide detailed descriptions of centrifugation parameters or final cellular composition (23, 24). In addition, only a minority of trials reported quantitative measures of cell counts, platelet concentration, or mononuclear cell fractions in the final product, which limits the ability to compare biological dosing across studies.

The volume and delivery strategy of the injected product also differed substantially. Intra-articular injections for knee osteoarthritis ranged from relatively small volumes of concentrated aspirate to larger preparations depending on the processing technique used (22, 28, 40). In surgical augmentation protocols—such as ACL reconstruction or rotator cuff repair—BMAC was frequently applied directly to grafts or repair sites rather than delivered as a free intra-articular injection (21, 29, 39). Similarly, studies combining BMAC with platelet-derived products introduced additional variability in the biological composition of the injected material (27, 37, 38).

### 3.6 Treatment failures and revision rates

Treatment failures and revision events were uncommon across trials. In rotator cuff repair, surgical failure rates were similar between groups despite improved structural integrity with BMAC (29). In ACL reconstruction, revision rates were low and comparable across BMAC and control arms (21, 41). In advanced osteonecrosis, BMAC did not reduce progression to arthroplasty compared with core decompression alone (35). No serious adverse events attributable to the biologic intervention were reported. Minor events, such as transient donor-site discomfort or localized pain, were infrequent and self-limited. Across knee, shoulder, and ACL studies, adverse events were comparable to controls, reinforcing the clinical tolerability of autologous bone marrow–based therapies.

### 3.7 Safety

Among trials that explicitly reported safety outcomes, BMAC and BMA demonstrated generally favorable safety profiles, with no serious adverse events directly attributed to the biologic intervention. Reported adverse events were predominantly mild and transient, including post-procedural pain, swelling, effusion, or donor-site discomfort, although safety reporting was incomplete in several trials. Trials in knee osteoarthritis reported low rates of minor complications (28, 40). Rotator cuff and ACL studies reported only self-limited musculoskeletal complaints (21, 29, 31, 38, 39) In osteonecrosis, Hauzeur et al. (35) described slightly higher donor-site tenderness in the BMAC group, without severe complications. Injection-related infections, neurovascular injuries, or systemic adverse events were rare.

Safety outcomes were summarized in a Supplementary Table S1, which compiles adverse events reported across all included randomized controlled trials. Across studies, most complications were mild and transient, including donor-site discomfort, temporary post-procedural pain, or local swelling. No serious adverse events directly attributable to BMA or BMAC were reported.

### 3.8 Subgroup effects

Several RCTs identified factors associated with differential treatment response. In knee osteoarthritis, Boffa et al. (22) reported greater clinical benefit in early-stage disease (Kellgren–Lawrence grades 1–2), a finding supported by complementary non-randomized evidence. In rotator cuff pathology, Centeno et al. (27, 38) suggested more robust structural healing in patients with smaller baseline tears.

Several trials evaluated BMA/BMAC as part of combination or augmentation protocols rather than as a stand-alone intervention. These included studies combining BMAC with platelet-derived products or using BMAC as a biologic adjunct during surgical procedures such as rotator cuff repair or ligament reconstruction. For example, Centeno et al. (27, 38) and Centeno et al. (37) administered BMAC together with platelet-derived products in the treatment of rotator cuff pathology and non-surgical ACL tears, reporting improvements in pain, functional outcomes, and imaging parameters. Similarly, surgical trials in rotator cuff repair and ACL reconstruction used BMAC as an augmentation strategy applied to grafts or repair sites rather than as an isolated biological intervention (21, 29, 39, 41). In these contexts, the biological preparation was integrated into broader therapeutic strategies that included mechanical repair or rehabilitation protocols. Consequently, the observed clinical improvements should be interpreted within the framework of multimodal treatment approaches rather than attributed exclusively to the isolated biological activity of BMA/BMAC.

### 3.9 Risk of bias

Overall, the methodological quality of the included trials was acceptable to good, with most studies classified as having low risk of bias or some concerns, and no trial judged to be at high risk of bias across multiple domains (Figure 2). Several trials, particularly those employing double-blind designs and multicenter methodologies, demonstrated robust randomization procedures and appropriate allocation concealment, resulting in a low risk of bias in the randomization domain (21, 22, 35, 40). Trials rated as having some concerns in this domain typically lacked detailed reporting of sequence generation or allocation concealment rather than presenting evidence of systematic bias.

**Figure 2:**
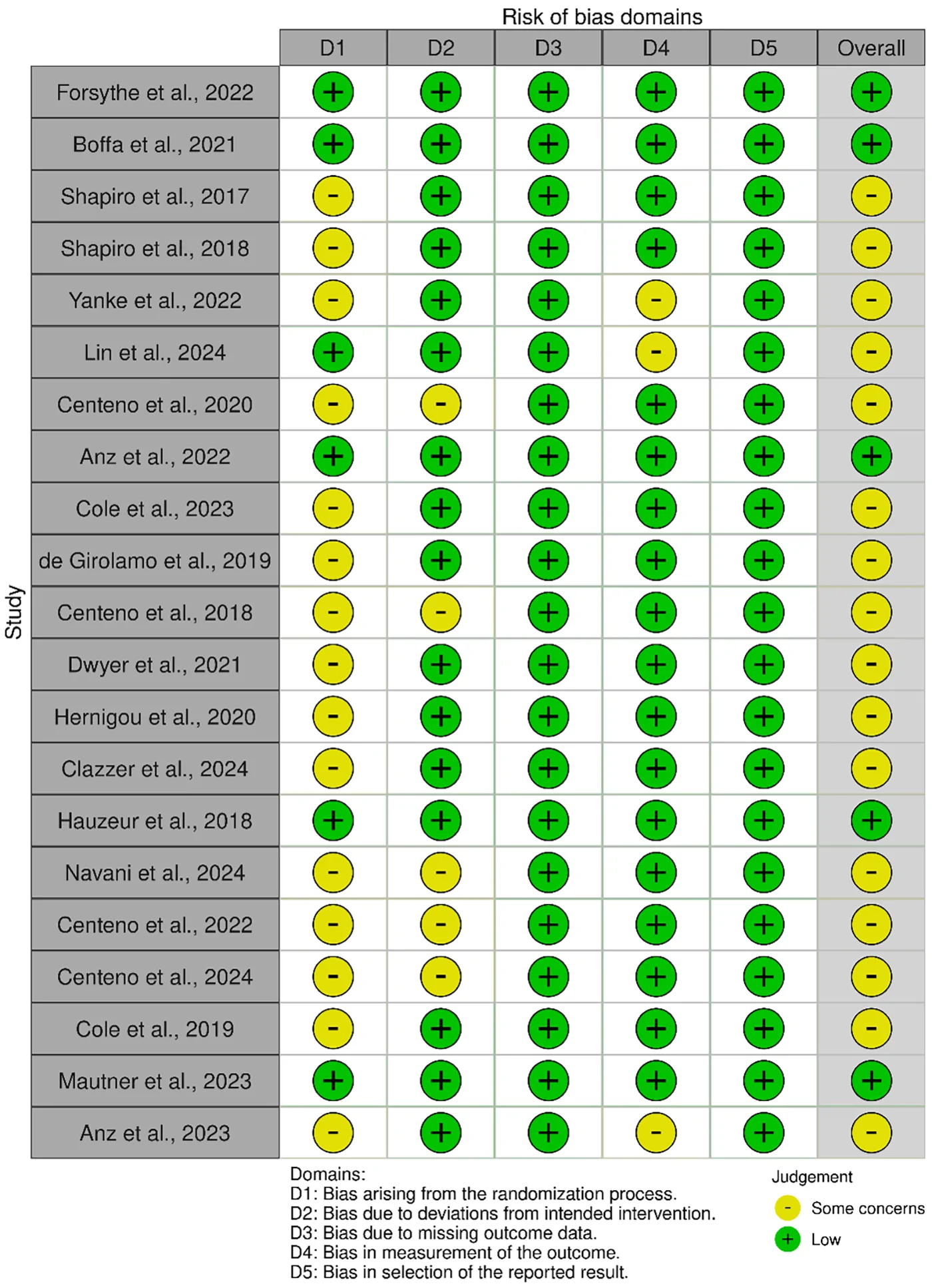
Risk-of-bias assessment of the included randomized trial reports using the Cochrane Risk of Bias 2 (RoB 2) tool. The assessment summarizes judgments across the five RoB 2 domains: bias arising from the randomization process, bias due to deviations from intended interventions, bias due to missing outcome data, bias in measurement of the outcome, and bias in selection of the reported result. Overall judgments were classified as low risk of bias, some concerns, or high risk of bias.

Bias due to deviations from intended interventions was generally low. Most studies maintained protocol adherence and reported outcomes consistent with the assigned interventions. Intention-to-treat analyses were performed when appropriate, particularly in larger pragmatic trials (40). In studies involving surgical augmentation or crossover designs, some concerns were noted, reflecting the inherent challenges of blinding participants and clinicians in orthobiologic and surgical trials, rather than demonstrable deviations likely to influence outcomes (27, 29, 38, 39).

Missing outcome data were minimal across trials, with high follow-up rates and balanced attrition between intervention and comparator groups. As a result, this domain was judged as having a low risk of bias in the majority of studies (22, 32, 40). When losses to follow-up occurred, they were generally well documented and unlikely to materially affect the direction or magnitude of treatment effects.

Outcome measurement was considered robust in most trials, as validated patient-reported outcome measures—such as the Visual Analog Scale (VAS), WOMAC, IKDC, DASH, KOOS, and ODI—were consistently employed, alongside standardized imaging protocols for structural assessment. Several trials reported blinding of outcome assessors, particularly for MRI-based endpoints (21, 39). Some studies were rated as having some concerns in this domain due to incomplete reporting of assessor blinding or reliance on subjective outcomes in partially unblinded settings.

Selective reporting bias was judged to be low in most studies. Primary outcomes were generally prespecified and reported in accordance with trial protocols or registration records when available (23, 24, 40). A small number of trials were rated as having some concerns due to limited access to preregistered protocols or insufficient clarity regarding outcome prioritization, rather than evidence of selective omission of unfavorable results.

Importantly, trials reporting favorable clinical or structural outcomes with BMA/BMAC were not systematically associated with higher risk of bias, supporting the internal validity of the observed effects.

## 4 Discussion

This systematic review of randomized controlled trials provides robust evidence that BMA and BMAC are safe and clinically effective in selected orthopedic contexts, with consistent signals of pain relief, functional improvement, and, in specific indications, structural benefit. Rather than functioning as a universal replacement for established therapies, BMA/BMAC appear best positioned as biologically active adjuncts capable of modulating inflammation, supporting tissue repair, and enhancing recovery—particularly when applied early in the disease course or in focal structural pathology.

Across RCTs, pain reduction was consistently observed in both intervention and control groups, suggesting that BMA/BMAC may exert clinically relevant analgesic effects but does not universally outperform conventional therapies. In knee osteoarthritis, BMAC demonstrated more durable pain improvement than hyaluronic acid in selected patients with milder disease (22), whereas direct comparison with PRP showed similar clinical improvement through 24 months (28). These findings align with contemporary evidence suggesting that biologic therapies may be more effective in milder degenerative phenotypes, where residual regenerative capacity remains, than in advanced structural disease (42).

In rotator cuff pathology, RCTs demonstrated a divergence between functional recovery and pain outcomes. While Centeno et al. (27, 38) showed superior pain and functional improvements with BMAC compared to exercise therapy, trials involving surgical augmentation reported comparable patient-reported outcomes regardless of biologic use (29, 39). This discrepancy may reflect a ceiling effect of surgery on pain and function, masking incremental benefits of biologic augmentation, a phenomenon previously described in cartilage and tendon repair trials (43, 44).

The interpretation of efficacy signals is further complicated by the use of combination therapies in several randomized trials. In particular, studies combining BMAC with platelet-derived products reported favorable outcomes in tendon and ligament injuries, suggesting potential synergistic biological effects mediated by the interaction between bone marrow–derived progenitor cells and platelet-derived growth factors (27, 37, 38). Platelet-derived products may enhance the local microenvironment by increasing concentrations of growth factors involved in angiogenesis, cellular recruitment, and extracellular matrix remodeling, which may amplify the regenerative signaling associated with bone marrow aspirate preparations.

These methodological differences may influence the biological potency of the therapy, as variations in cellular concentration, growth factor content, and delivery technique could affect the regenerative and immunomodulatory properties of the final product. Consequently, the heterogeneity in preparation methods represents an important source of variability across trials and may partially explain differences in reported efficacy signals. Therefore, treatment effects should be interpreted with caution, recognizing that variability in BMA/BMAC preparation protocols and dosing strategies may contribute to differences in clinical outcomes across studies.

Similarly, in surgical contexts such as rotator cuff repair and anterior cruciate ligament reconstruction, BMAC was frequently applied as a biologic augmentation to the repair site or graft interface rather than delivered as a stand-alone therapy (21, 29, 39, 41). In these studies, improvements in structural integrity, graft maturation, or reduced retear rates may reflect the interaction between mechanical stabilization and biologic stimulation of tissue repair. This multimodal environment complicates attribution of clinical benefits to a single component of the intervention. Therefore, the available evidence should be interpreted as supporting the role of BMA/BMAC as a biologic adjunct that may enhance tissue healing when combined with appropriate mechanical or rehabilitative strategies, rather than as an isolated treatment capable of independently driving clinical recovery in all orthopedic contexts.

One of the most striking findings of this review is the frequent dissociation between imaging-based healing and clinical outcomes. In ACL reconstruction, some RCTs demonstrated imaging evidence suggestive of accelerated ligamentization with BMAC (21, 45). However, multiple high-quality trials failed to confirm consistent improvements in graft maturation, volume, or tendon–bone interface characteristics (26, 41). This variability underscores ongoing challenges in interpreting MRI biomarkers as surrogates for functional recovery, especially given the complex temporal dynamics of graft remodeling (46–49).

In osteochondral and focal cartilage procedures, structural findings were mixed. De Girolamo et al. (30) demonstrated satisfactory cartilage repair in both AMIC and AMIC + BMAC groups, with an early clinical advantage for the augmented approach. In osteochondral allograft transplantation, Yanke et al. (25) found comparable osseous integration between groups but a lower subsequent reoperation rate after BMAC-treated grafts.

Conversely, in knee osteoarthritis and advanced osteonecrosis, imaging outcomes were largely neutral. Placebo-controlled trials showed no evidence of cartilage regeneration in osteoarthritis (23), and advanced-stage osteonecrosis did not respond structurally to BMAC compared with core decompression alone (35). These results reinforce the concept that orthobiologic interventions may not reverse established structural collapse or diffuse degenerative processes (11, 50).

A critical contribution of this review is the identification of consistent subgroup effects across RCTs. Multiple trials demonstrated that the clinical benefit of BMAC is moderated by disease severity, with early-stage osteoarthritis responding more favorably than advanced disease (22). In ACL reconstruction, BMAC may exert its effects through modulation of local inflammatory or reparative processes rather than direct enhancement of ligament healing alone (51). These findings highlight the importance of precision-based patient selection and argue against indiscriminate application of BMA/BMAC across all orthopedic conditions. Instead, they support a stratified approach that considers biological milieu, mechanical environment, and disease stage.

Importantly, safety outcomes across all RCTs were favorable. No serious adverse events directly attributable to BMA/BMAC were reported, and minor complications—such as transient pain or donor-site discomfort—were infrequent and self-limited (22, 35, 40). These findings are consistent with previous safety-focused analyses and support the overall tolerability of autologous bone marrow–based interventions when performed under appropriate clinical standards (33).

From a clinical perspective, the results suggest that BMA/BMAC should not be viewed as a universal alternative to established therapies but rather as a selective adjunct in well-defined scenarios. Evidence is strongest for focal cartilage defects, selected rotator cuff tears, early-stage knee osteoarthritis, and ACL reconstruction in the presence of concomitant pathology. In contrast, its routine use in advanced degenerative disease or as a substitute for effective conventional treatments is not supported by high-level evidence.

Among studies classified as having “some concerns,” the most frequent methodological issues were related to incomplete reporting of allocation concealment procedures, limited description of randomization methods, and insufficient clarity regarding adherence to pre-specified outcome protocols. These limitations were observed in several trials where the methods section did not fully specify the random sequence generation process or the procedures used to ensure adequate concealment of treatment allocation prior to enrollment (23, 24, 28, 34). Such reporting limitations make it difficult to fully exclude the possibility of selection bias, particularly in studies with relatively small sample sizes or single-center designs.

Another common source of concern involved deviations from intended interventions and selective outcome reporting. In some trials, outcome assessment protocols were not clearly linked to pre-registered trial protocols or predefined statistical analysis plans (28, 34, 36). This lack of transparency may increase the risk of reporting bias, as it becomes more difficult to determine whether all planned outcomes were reported consistently across study groups.

Blinding procedures also represented a challenge in several surgical or procedure-based trials. In studies evaluating BMAC as an adjunct to surgical repair—such as anterior cruciate ligament reconstruction or rotator cuff repair—blinding of surgeons and participants was often not feasible due to the nature of the intervention (21, 29, 39, 41). The absence of blinding in such contexts may introduce performance bias and potentially influence patient-reported outcomes or postoperative management.

Despite these limitations, the majority of trials demonstrated generally adequate methodological rigor, including randomized allocation, prospective follow-up, and standardized outcome assessment. Importantly, no systematic patterns of bias were identified that would consistently favor BMA/BMAC interventions over comparator treatments. Therefore, while some methodological uncertainties remain, these concerns primarily affect the precision of estimated treatment effects rather than fundamentally altering the overall interpretation of the evidence.

This review has several limitations. Although restricted to RCTs, substantial heterogeneity remained in cell preparation methods, delivery techniques, outcome measures, and follow-up durations, precluding meta-analysis. Cell counts and biological composition were inconsistently reported, limiting dose–response interpretation. Additionally, some long-term RCTs relied on imaging or clinical endpoints that may not fully capture patient-centered outcomes. Finally, regulatory and reporting variability across regions may influence external validity.

Emerging applications of BMA/BMAC also include hip preservation surgery, particularly biologic augmentation during arthroscopic acetabular labral repair and treatment of associated chondral pathology. Martin et al. (52) compared 62 patients undergoing acetabular labral repair with BMAC augmentation with 62 controls and found no significant overall difference in postoperative iHOT-33 scores; however, among patients with moderate cartilage damage (Outerbridge grades 2–3), BMAC was associated with higher iHOT-33 scores at 12 months (78.6 vs. 69.2; *P* = .035) and 24 months (82.5 vs. 69.5; *P* = .030). Day et al. (53) similarly reported clinically significant improvement after hip arthroscopy with concomitant BMAC in patients with symptomatic labral tears and early degenerative changes, although outcomes were broadly comparable with those of a matched nonarthritic cohort. More recently, a systematic review and meta-analysis of four controlled studies including 315 patients found no statistically significant pooled advantage of BMAC augmentation in iHOT-33 outcomes at either 12 (*P* = .14) or 24 months (*P* = .30) (54). Thus, although these findings suggest a potential role for BMAC in selected patients with concomitant chondral damage, the available hip literature remains predominantly non-randomized, and adequately powered randomized trials are required before routine use can be recommended.

Future RCTs should prioritize standardized reporting of BMA/BMAC composition, including cellular concentration and biological product characteristics, stratification by disease phenotype, and integration of multimodal outcomes combining patient-reported measures with validated imaging and biomechanical endpoints. Comparative effectiveness studies against optimized rehabilitation protocols and established biologic therapies, together with cost-effectiveness analyses, are also needed to better define the role of BMA/BMAC within contemporary orthopedic care. Further adequately powered randomized studies are particularly warranted in hip preservation surgery, including acetabular labral repair, femoroacetabular impingement, focal chondral pathology, and early hip osteoarthritis.

In conclusion, the totality of evidence suggests that BMA/BMAC should be considered targeted, mechanism-driven interventions rather than empirical treatments. Strongest support exists for early-stage knee osteoarthritis, focal cartilage defects, selected rotator cuff pathology, non-surgical ligament injuries, and ACL reconstruction in biologically vulnerable joints. Conversely, expectations should be tempered in advanced degenerative disease, where structural damage may exceed the reparative capacity of biologic augmentation.

## Data Availability

The raw data supporting the conclusions of this article will be made available by the authors, without undue reservation.
