# Supplementary material for: Efficacy and safety of bone marrow aspirate and bone marrow aspirate concentrate in orthopedic conditions: a systematic review of randomized controlled trials
Source: Front Surg. 2026 Oct 2;13:1932090. doi: 10.3389/fsurg.2026.1932090 (PMC13671967; doi:10.3389/fsurg.2026.1932090)
Supplement: Supplementary file 1 [file Table1.docx]

**Table S1.** Adverse events reported across randomized controlled trials evaluating BMA/BMAC in orthopedic conditions

| **Study report(s)** | **Condition** | **BMA/BMAC intervention** | **Comparator** | **Adverse events / safety findings as reported** | **Safety interpretation** |
| --- | --- | --- | --- | --- | --- |
| Forsythe et al.^21^ | ACL reconstruction | BMAC graft augmentation | Standard ACL reconstruction | NR; adverse events or complications were not separately quantified as a dedicated outcome | BMAC-specific safety frequency not extractable |
| Boffa et al.^22^ | Knee osteoarthritis | Intra-articular BMAC | Hyaluronic acid | No severe AEs; mild AEs occurred in 7.1% of BMAC-treated knees and 5.4% of HA-treated knees (P=n.s.) | Comparable minor AE frequency; no severe safety signal |
| Shapiro et al.^23,24^ | Bilateral knee osteoarthritis | Intra-articular BMAC | Saline placebo | No serious AEs. Early postprocedural effusion occurred in 58% of BMAC-treated versus 25% of placebo-treated knees, decreasing to 12% versus 8% at 6 months. One transient episode of warmth occurred; no erythema was reported | Transient local reactions; no serious safety signal |
| Yanke et al.^25^ | Osteochondral allograft transplantation | BMAC applied to graft | Standard transplantation | NR; adverse events were not separately quantified as a dedicated safety outcome | BMAC-specific AE rate not extractable |
| Lin et al.^26^ | ACL reconstruction | BMAC + PRP augmentation | PRP / standard ACL reconstruction | Clinical AEs were not separately quantified; peri-tunnel edema was reported as an MRI finding rather than a clinical AE | Clinical safety frequency not separately extractable |
| Centeno et al.^27,38^ | Rotator cuff / supraspinatus tears | BMC + PRP + platelet lysate | Exercise therapy | No serious adverse events were reported through 2 years | No serious safety signal |
| Anz et al.^28^ | Knee osteoarthritis | Intra-articular BMC | PRP | NR; adverse events were not separately quantified in the 24-month report | Comparative safety estimate not extractable |
| Cole et al.^29,39^ | Rotator cuff repair | cBMA augmentation | Standard repair | Complications occurred in 0/46 cBMA-treated patients versus 1/45 controls (2.2%; P=.495); no other documented complications were reported | No excess complication signal with cBMA augmentation |
| de Girolamo et al.^30^ | Focal knee chondral defects | AMIC + BMAC | AMIC | One case of arthrosynovitis occurred in the standard AMIC group; no other adverse events or complications were recorded | No reported AE attributable to BMAC augmentation |
| Centeno et al.^31^ | Knee osteoarthritis | BMC + platelet products | Exercise therapy | No serious adverse events were reported | No serious safety signal |
| Dwyer et al.^32^ | Glenohumeral osteoarthritis | Intra-articular nonconcentrated BMA | Corticosteroid | NR; adverse events or complications were not separately quantified as a dedicated outcome | Safety frequency not extractable |
| Hernigou et al.^33^ | Knee osteoarthritis | Subchondral BMC | Contralateral TKA | NR; adverse events were not separately extractable from the published trial report | Safety not separately quantified |
| Clazzer et al.^34^ | Knee osteoarthritis | Intra-articular BMA within injection/block protocol | Corticosteroid + genicular nerve block | NR; no dedicated adverse-event analysis or treatment-arm AE frequency was reported | Safety not separately quantified |
| Hauzeur et al.^35^ | Osteonecrosis of the femoral head, ARCO stage 3 | Core decompression + BMAC | Core decompression + saline | No serious AEs. Greater-trochanter pain occurred in 4 cases (1 BMAC); iliac-crest pain in 2 cases (both BMAC); transient fever <24 h in 2 cases (both BMAC); nausea in 2 cases (1 BMAC) | Reported events were minor/transient; no serious safety signal |
| Navani et al.^36^ | Discogenic low back pain | Intradiscal BMC | Intradiscal PRP / placebo | No local injury from bone marrow extraction, local or systemic infection, neurologic injury, or hospitalization was reported. Temporary low back pain after disc injection was the most frequently reported AE and resolved within 3–5 days | Transient procedure-related pain; no serious safety signal |
| Centeno et al.^37^ | Non-surgical ACL tears | BMC + PRP + platelet lysate | Exercise therapy | No serious AEs. Reported events included 1 knee effusion requiring aspiration, 1 temporary paresthesia associated with brace use, and 1 case of residual BMA-site pain that resolved spontaneously | Minor and self-limited AEs; no serious safety signal |
| Mautner et al.^40^ | Knee osteoarthritis | Intra-articular BMAC | Corticosteroid, adipose-derived SVF, or UCT-MSC | No procedure-related serious AEs, symptomatic infections, or allergic reactions. Among BMAC recipients, postprocedural contusion occurred in 12.2% and hematoma in 2.9% | No serious safety signal; minor local procedural events occurred |
| Anz et al.^41^ | ACL autograft reconstruction | Amnion collagen matrix + BMAC | Standard ACL reconstruction | No adverse events were reported through 2 years | No AE signal; findings apply to the combined amnion+BMAC intervention |

**Abbreviations:** ACL, anterior cruciate ligament; AE, adverse event; AMIC, autologous matrix-induced chondrogenesis; ARCO, Association Research Circulation Osseous; BMA, bone marrow aspirate; BMAC/BMC, bone marrow aspirate concentrate/bone marrow concentrate; cBMA, concentrated bone marrow aspirate; HA, hyaluronic acid; MRI, magnetic resonance imaging; PRP, platelet-rich plasma; SAE, serious adverse event; SVF, stromal vascular fraction; TKA, total knee arthroplasty; UCT-MSC, umbilical cord tissue-derived mesenchymal stromal cells, NR, not reported or not separately extractable as a dedicated adverse-event result. Reoperations and treatment failures were not classified as adverse events unless explicitly reported.
